# Spatial Induction in Color Scission

**DOI:** 10.1177/20416695211000364

**Published:** 2021-03-12

**Authors:** Zhehao Huang, Qasim Zaidi

**Affiliations:** Graduate Center for Vision Research, State University of New York, New York, United States

**Keywords:** transparency, color, scission, induction

## Abstract

An exception to the rule that only one color is seen at every retinotopic location happens when a bounded colored transparency or spotlight is seen on a differently colored surface. Despite the spectrum of the light from each retinotopic location being an inextricable multiplication of illumination, transmission, and reflectance spectra, we seem to be able to scission the information into background and transparency/spotlight colors. Visual cues to separating overlay and overlaid layers have been enumerated, but neural mechanisms that extract veridical colors for overlays have not been identified. Here, we demonstrate that spatial induction contributes to color scission by shifting the color of the overlay toward the actual color of the filter. By alternating filter and illumination spectra, we present naturalistic simulations where isomeric disks appear to be covered by filters/spotlights of near veridical colors, depending solely on the surrounding illumination. This previously unrecognized role for spatial induction suggests that color scission employs some general purpose neural mechanisms.

Seeing the color of a surface as separate from the color of an overlaying filter, spotlight, shadow, or fog (D’Zmura et al., 2000), is known as scission (Anderson & Khang, 2010). This ability facilitates judging the color of transparent volumes (Ennis & Doerschner, 2019), estimating illuminant color as an indicator of weather or time of day (Zaidi, 1998), and inferring surface colors (Brainard & Maloney, 2011). The disentangling problem is formally underdetermined if only the overlaid region is considered, because the spectral distribution coming from the overlaid image is the result of the illuminant spectrum transmitted through the spectrum of the intervening medium, and reflected from the spectrum of the surface, with both transmittance and reflectance leading to wavelength-by-wavelength multiplication of spectra. Scission, however, becomes a solvable problem when information from the surround is incorporated in the empirically established circumstance that (L, M, and S) cone absorptions from overlaid regions are three-dimensional diagonal transforms of cone absorptions from exposed regions, or equivalently an affine transform in cone-opponent space (Khang & Zaidi, 2002a, 2002b; Westland & Ripamonti, 2000), opening up the possibility that neural mechanisms could internalize this associational invariance and exploit it as a heuristic. Khang and Zaidi (2004) showed that perceived filter colors across different colored backgrounds were consistent with a model where observers matched the color transform across backgrounds. We discovered that the matching is less veridical on a constant background across different illumination spectra. To understand neural mechanisms involved in color scission, we designed novel *isomeric* stimuli. To our surprise, we discovered that classical color induction (Zaidi, 1999) is responsible in large part for setting the perceived color of the filter/spotlight, and we demonstrate that in this article with stills and videos.

In Figure 1A (click to activate video), the circular disks in the two left-most panels are physically identical but appear different. The Y_I_C_F_ panel shows a variegated achromatic background under an illuminant (subscript I) with the spectrum of a Kodak CC50 yellow (Y) filter (Figure 1E), with the light then passing once through a circular Kodak CC50 cyan (C) filter (subscript F). In the C_I_Y_F_ panel, the illuminant has the cyan filter’s spectrum and passes through a circular yellow filter. The lights coming to the eye from the two disks are identical because the spectrum of the light in both cases is the wavelength-by-wavelength multiplication (C × Y) of the yellow and cyan spectra, reflected from the same gray-level background. However, the filter color in C_I_Y_F_ appears yellower than in Y_I_C_F_, an example of partial color scission. The three left panels (W_I_C_F_ × Y_F_, W_I_C_F_, and W_I_Y_F_) show the C_F_ × Y_F_, C_F_ and Y_F_ filters under an equal-energy white (W) light. For convenience, we will call these the *essential* colors of the filters (Zaidi, 1998). The perceived color of the filter in Y_I_C_F_ is shifted from the essential color of the identical C_F_ × Y_F_ filter toward C_F_ but not completely. The perceived color of the filter in C_I_Y_F_ is also shifted away from C_F_ × Y_F_, but toward Y_F_, again incompletely. These *illusory* shifts in filter color are the first demonstrations of simultaneous color induction for simulated filters, showing that spatial induction contributes to color scission in naturalistic displays by making filters appear closer to their essential color. The perceived shift is in the color direction that is complementary to the surround color with respect to the center color as described by Krauskopf et al. (1986), consistent with color induction being mediated by higher order color mechanisms preferentially tuned to a wide variety of color directions, like those in extra-striate cortex (Zaidi & Conway, 2019).

**Figure 1.** A: Illuminant*-*filter transparency conditions on a variegated gray background, indicated by subscripts on color names. B: Opacity conditions with the same spatial and color distribution as (A). C: Illuminant-spotlight transparency conditions. D: Opacity conditions with the same spatial and color distribution as (C). E: Spectral distribution of Kodak CC50 cyan and yellow filters, and C × Y and C + Y. F: L, M, and S cone catches of materials under C × Y (black) and C + Y (gray) plotted against catches under C (diamonds) or Y (squares). Each diamond or square represents one material. Filled symbols represent catches from the filtered lights directly. Click to activate video.

Anderson and Khang (2010) demonstrated substantial color induction involving percepts of moving transparent layers and suggested that scission precedes induction. Ekroll and Faul (2013) went further and claimed that transparency underlies induction. Induction, however, happens even when no percept of transparency is possible, for example, inside a spatially uniform disc surrounded by drifting radial sine waves (Zaidi et al., 1992) or dynamic texture (Spehar et al., 1996). The panels in Figure 1B have the same spatial and color distributions as the panels above them, but when the video is activated, the initial disk is moved over the background, replacing transparency promoting X-junctions into T-junctions evoking a strong percept of a moving opaque patch (Khang & Zaidi, 2002a). C_I_Y_F_ again appears yellower than in Y_I_C_F_, showing that induction occurs even in the absence of transparency or scission, so we maintain that induction has primacy, and in displays that simulate naturalistic conditions, it shifts the perceived filter color toward its essential color.

The filter displays are naturalistic in the limited sense that the colors are computed using spectra of real objects and filters, and the optical calculations are decent approximations to physical reality. Another naturalistic way to obtain identical transparent disks is to shine circular colored spotlights (subscript S) on top of illuminated scenes (Figure 1C) so that spectra of overlays are the wavelength-by-wavelength addition of spotlight and illumination spectra. The spotlight in C_I_Y_S_ appears yellower than in Y_I_C_S_, despite the two disks being physically identical. The perceived spotlight colors are shifted toward their essential colors from the essential color of C_S_+Y_S_. The induced effect thus holds for spotlights too and for opaque patches with the same color distributions (Figure 1D). Spotlights have a less saturated appearance than filters because C_S_+Y_S_ is a less selective spectrum than C_F_ × Y_F_ (Figure 1E).

We tried many pairs of filters and found similar induced effects. As the C × Y and C + Y filters passed bands of wavelengths, to illustrate the effect for the opposite case of notch filters that block roughly the same band, we recreated the filter and spotlight conditions for red and blue Kodak CC50 filters (click on Figure 2 to activate video). The R&B effects are similar to C&Y.

**Figure 2.** A: Illuminant*-*filter transparency conditions on a variegated gray background, indicated by subscripts on color names. B: Opacity conditions with the same spatial and color distribution as (A). C: Illuminant-spotlight transparency conditions. D: Opacity conditions with the same spatial and color distribution as (C). E: Spectral distribution of Kodak CC50 red and blue filters, and R × B and R + B. F: L, M, and S cone catches of materials under R × B (black) and R + B (gray) plotted against catches under R (diamonds) or B (squares). Each diamond or square represents one material. Filled symbols represent catches from the filtered lights directly. Click to activate video.

To quantify the direction and magnitude of the induced color shifts for the filters, observers were shown adjacent pairs of displays on a 48° × 27° (1,920 × 1,080 pixels) calibrated screen covered with oriented elliptical patches (axis lengths 0.85° to 1.56°) with uniform spectral reflectance. One half of the display simulated a circular 7° disk with the (A_F_ × B_F_) filter spectrum surrounded by A_I_ or B_I_ illuminated ellipses, and the other of a similar disk with the filter spectrum ( $(A_{F}\times B_{F})-\;kA_{F}+kB_{F}$ ) surrounded by W illumination, where the spectrum of the filter could be adjusted by k in the range [−1,1]. Observers were asked to match the colors of the two filters, ignoring small differences in brightness. The sign of k gave the direction of the perceived shift and 2 k gave the magnitude. Interleaved in the experiment were matches to the moving opaque patches with the same color conditions. Averaged over three observers, the perceived shift in Y_I_C_F_ was 0.428 and 0.056 from Y_F_ × C_F_ toward C_F_ for the transparent and patch conditions, respectively. The shift in C_I_Y_F_ was 0.268 and 0.026 toward Y_F_. The shift in R_I_B_F_ was 0.128 and 0.072 from R_F_ × B_F_ toward B_F_. The shift in B_I_R_F_ was 0.140 and 0.104 toward R_F_. These results clearly show that induction moves the perceived color of the filter toward the color under neutral illumination, thus contributing to color scission, and although induction is greater under transparency than opacity percepts, there is reliable induction even with cues against transparency.

For measuring induction for simulated spotlights, one half of the display simulated a circular 7° disk with the (A_S_+B_S_) spotlight spectrum surrounded by A_I_ or B_I_ illuminated ellipses, and the other of a similar disk with the spotlight spectrum ( $(A_{S}+B_{S})-kA_{S}+kB_{S}$ ) surrounded by W illumination. The perceived shift in Y_I_C_S_ was 0.428 and 0.042 from Y_S_ + C_S_ toward C_S_ for the transparent and patch conditions, respectively. The shift in C_I_Y_S_ was 0.314 and 0.284 toward Y_S_. The shift in R_I_B_S_ was 0.134 and 0.102 from R_S_+B_S_ toward B_S_. The shift in B_I_R_S_ was 0.130 and 0.078 toward R_S_. Measured induction for spotlights is also always in the direction toward the essential color of the spotlight over neutral illumination.

As the scission is partial, we checked to see whether there is sufficient information in the displays for a model to achieve complete scission. Figure 1F shows that the L, M, and S cone catches from the background ellipses in the disks under C × Y and C + Y are almost perfectly correlated with catches from ellipses in the surround under C and Y. The slopes are also equal to the ratio of cone catches from the filter and illuminant spectra (filled symbols). Figure 2F shows similar perfect correlations for B and R. Khang and Zaidi (2004) showed that a visual system could match filter colors across backgrounds by equating ratios of spatially integrated cone catches from disks and surrounds, confirmed for volumetric transparency by Ennis and Doerschner (2019). We confirmed that the application of this model yielded essential Y, C, B, and R colors instead of the perceived colors, revealing that color scission is not perfect even when there is sufficient information.

The ability to match a filter color across backgrounds is often used to measure the degree of color scission. This article demonstrates that spatial induction contributes to color scission for filters and spotlights by shifting their perceived colors toward their essential colors. Why the color shift is seen predominantly on the overlay separated from the overlaid segment, could be modeled by simplicity or constancy assumptions about the background, but that is not the only possibility, so we hope our demonstrations will spur critical examination of this issue.
